# Bibliometric and visual analysis of human microbiome—breast cancer interactions: current insights and future directions

**DOI:** 10.3389/fmicb.2024.1490007

**Published:** 2024-12-09

**Authors:** Yi Zhou, Min Jiang, Xiaoyu Li, Ke Shen, Hui Zong, Qing Lv, Bairong Shen

**Affiliations:** Department of Breast Surgery and Institutes for Systems Genetics, Frontiers Science Center for Disease-Related Molecular Network, West China Hospital, Sichuan University, Sichuan, China

**Keywords:** breast cancer, microbiome, intratumor bacteria, microbiome–host interaction, bibliometric analysis

## Abstract

The composition of the gut microbiome differs from that of healthy individuals and is closely linked to the progression and development of breast cancer. Recent studies have increasingly examined the relationship between microbial communities and breast cancer. This study analyzed the research landscape of microbiome and breast cancer, focusing on 736 qualified publications from the Web of Science Core Collection (WoSCC). Publications in this field are on the rise, with the United States leading in contributions, followed by China and Italy. Despite this strong output, the centrality value of China in this field is comparatively low at ninth, highlighting a gap between the quantity of research and its global impact. This pattern is repetitively observed in institutional contributions, with a predominance of Western institutes among the top contributors, underscoring a potential research quality gap in China. Keyword analysis reveals that research hotspots are focused on the effect of microbiome on breast cancer pathogenesis and tumor metabolism, with risk factors and metabolic pathways being the most interesting areas. Publications point to a shift toward anti-tumor therapies and personalized medicine, with clusters such as “anti-tumor” and “potential regulatory agent” gaining prominence. Additionally, intratumor bacteria studies have emerged as a new area of significant interest, reflecting a new direction in research. The University of Helsinki and Adlercreutz H are influential institutions and researchers in this field. Current trends in microbiome and breast cancer research indicate a significant shift toward therapeutic applications and personalized medicine. Strengthening international collaborations and focusing on research quality is crucial for advancing microbiome and breast cancer research.

## 1 Background

The human body hosts trillions of microorganisms, including bacteria, viruses, fungi, and other microbes, collectively known as the human microbiome (Baquero and Nombela, 2012). These microbes inhabit various body sites, such as the skin, mouth, gut, and reproductive tract, where they play important roles in maintaining health and regulating various physiological processes (Human Microbiome Project Consortium, 2012; Lozupone et al., 2012). An emerging area of research focuses on the microbiome’s impact on diseases, including breast cancer (Urbaniak et al., 2016). Breast cancer is the most prevalent cancer among women worldwide, and its development is influenced by multiple factors, including genetic predisposition, lifestyle choices, and environmental exposures (Shagisultanova et al., 2022; Sung et al., 2021). Recent studies have begun exploring the potential connection between the microbiome and breast cancer risk, development, and treatment outcomes (Nearing et al., 2023).

In breast cancer research, differences in microbiome composition have been observed between breast cancer patients and healthy individuals (Hou et al., 2021). These differences suggest that the microbiome may contribute to the development or progression of breast cancer. Animal model studies further support this idea, as altering gut microbiome composition has been shown to influence breast tumor growth and metastasis (Sampsell et al., 2022). Proposed mechanisms linking the microbiome to breast cancer include the modulation of estrogen metabolism, inflammation, immune responses (Lee et al., 2022; Arnone et al., 2024; Yue et al., 2023), and the production of metabolites that can affect tumor growth and response to treatment (Mikó et al., 2018; Chlebowski et al., 2024). Understanding these mechanisms is crucial for developing targeted interventions that may help prevent or treat breast cancer by modulating the microbiome. Although research in the field of microbiome and breast cancer is still in its early stages, it shows promise in identifying novel biomarkers, therapeutic targets, and prevention strategies. Given that many mechanisms of action remain unclear, further studies are needed to clarify the complex interactions between the microbiome and breast cancer, potentially leading to significant advancements in disease understanding and management, including microbiome-based therapies as adjuncts in breast cancer treatment.

Bibliometrics provides a systematic method for evaluating scientific articles in a specific research field by qualitative and quantitative analysis, which provides an objective and intuitive understanding of scientific research trends (Wu et al., 2024). Bibliometrics analysis is valuable for assessing the current status and future directions of disciplinary research. Additionally, bibliometrics allows us to understand collaborations among authors, institutions, and countries, thereby assessing their academic contributions to a particular research field (Khalil and Gotway Crawford, 2015). However, no bibliometric study currently examines the relationship between the human microbiome and breast cancer.

Therefore, conducting a bibliometric analysis to highlight current research hotspots and fronts is essential for understanding the direction of research in this field and addressing existing gaps. This study aims to conduct a systematic bibliometric analysis of research on the microbiome and breast cancer from the year 2000 to the present. Through this analysis, we aim to comprehensively present the latest advancements and future directions in the field, enabling researchers to quickly grasp the current landscape and development trends, fostering scientific progress, and ultimately benefiting patients.

## 2 Methods

### 2.1 Data source and search strategy

**Figure 1:**
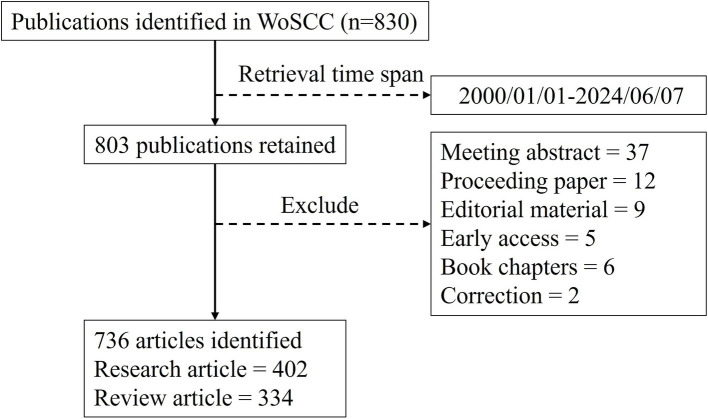
Flow chart of the publication retrieval process.

All published literature was retrieved from the WoSCC using a specified search strategy, which is detailed in the Supplementary material. The literature search covered publications from 1 January 2000 to 7 June 2024 and included articles and reviews as the document type. The retrieved documents were exported from the WoSCC as plain text files with full records and cited references for subsequent bibliometric analysis. To ensure a comprehensive and accurate retrieval process, the literature search was conducted independently by our team on 7 June 2024. All extracted publications were manually reviewed to verify their uniqueness and correct classification. The complete retrieval workflow is illustrated in Figure 1.

### 2.2 Literature analysis

The CiteSpace.6.3.R2 (64-bit) Advanced, Microsoft Excel, and Notepad++ were applied for data analysis and management. CiteSpace is used for providing a detailed summary analysis of annual publications by number, country, institution, author, keyword, and highly cited article. Microsoft Excel and Notepad++ are used for data management. The R package “plotRCS (v 0.1.5)” was used for restricted cubic spline (RCS) analysis.

## 3 Results

### 3.1 Annual publications and the trends in publications

**Figure 2:**
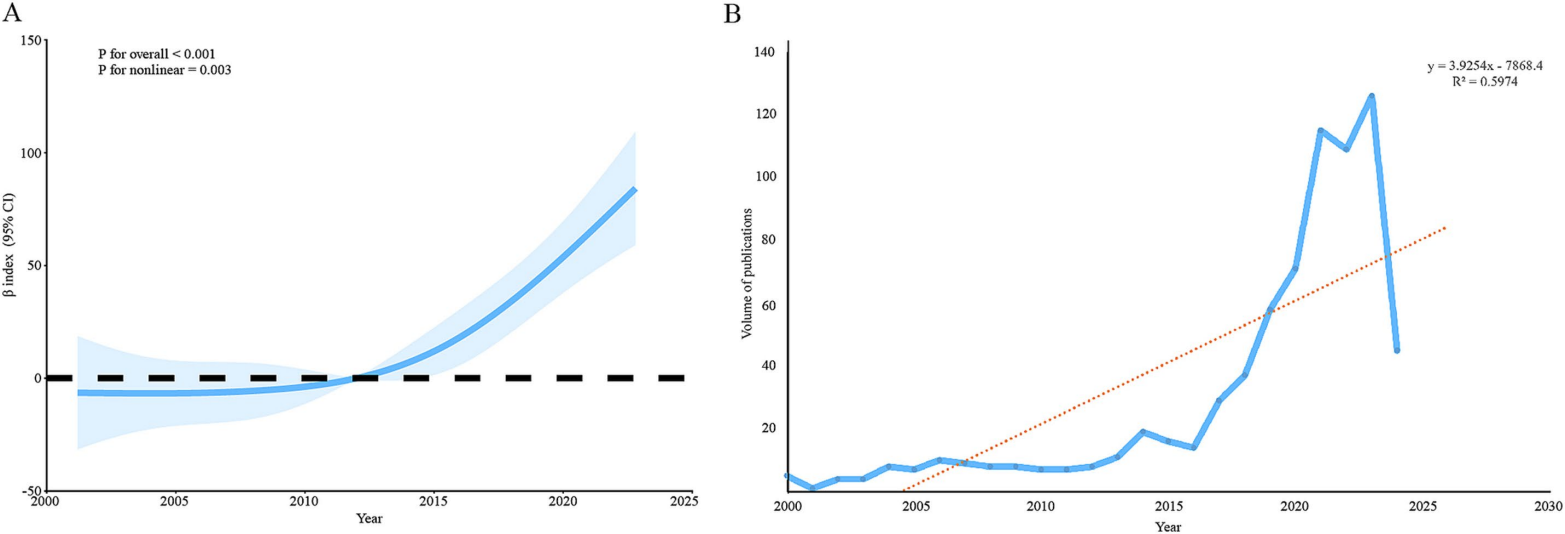
Annual publication volume trend on the microbiome and breast cancer. The analysis using restricted cubic spline curves **(A)** and a linear model **(B)** suggests that the number of publications has gradually increased over time.

A total of 736 items were retrieved from the WoSCC database, comprising 402 research articles and 334 review articles published between 2000 and 7 June 2024. The data indicate a consistent growth in publication numbers over the years, peaking at 126 articles in 2023. The results of the nonlinear RCS analysis showed a statistically significant overall trend (*p*-value <0.001) and a significant nonlinear component (*p*-value = 0.003), suggesting that the publication volume is likely to continue increasing in 2024. Furthermore, the linear trend analysis, with an R-squared value of 0.5974 (Figure 2), supports the observation of a continuous upward trend in publication volume.

### 3.2 Contribution in different countries, institutions, and the authors

A total of 73 countries, 447 institutions, and 612 authors have contributed to this research topic. The network graph visually represents this information, with node sizes indicating the publication volume of each country. Larger nodes correspond to countries with a higher number of publications in the field. Notably, countries with a centrality value exceeding 0.1 are marked with a purple circle around their nodes on the network map, signifying their significant influence within the research network. Centrality is a measure used to assess the importance or influence of a node within a network, helping to identify significant contributions made by specific countries within this research field.

**Figure 3:**
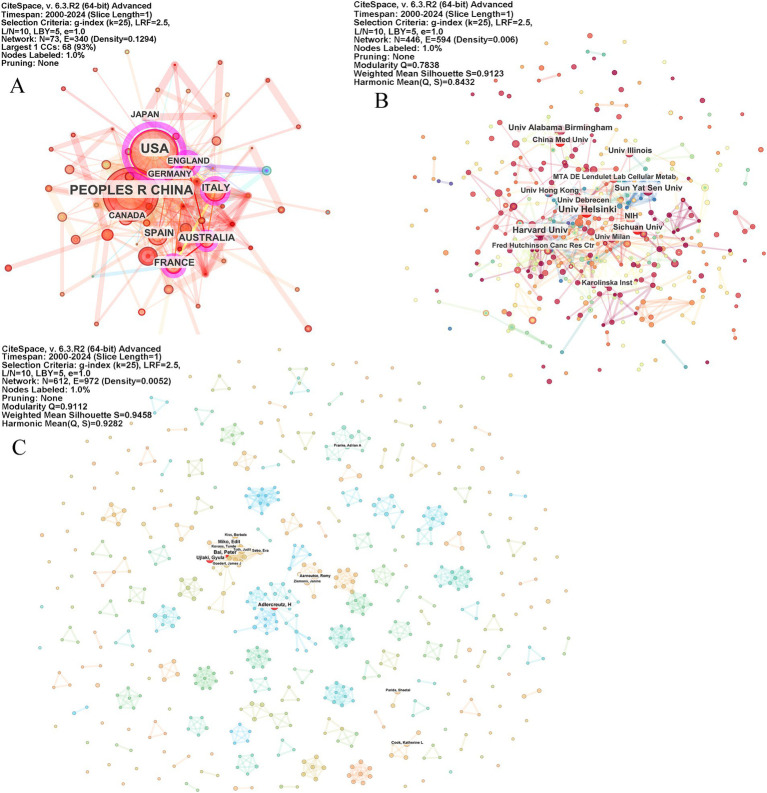
Country, institute, and author collaboration network on microbiome and breast cancer. The network graph visualizes the contributions of countries **(A)**, institutions **(B)**, and authors **(C)**, with node sizes indicating the publication volume of the contributors. Countries with a centrality value exceeding 0.1 are highlighted with a purple circle around their nodes on the network map. The parameters of the CiteSpace analysis are displayed in the top right corner of each figure.

Table 1 and Figure 3A illustrate the top 10 countries contributing to publication output in this field. The United States, China, Italy, Spain, and Australia are the leading contributors, with the USA accounting for 23.18% and China accounting for 17.43% of the total publications. Together, these two countries contribute to over 40% of the research output in this area. However, in terms of centrality, which indicates the significance of contributions and collaboration between countries, the USA ranks first with a score of 0.63, while China ranks eighth with a score of 0.07.

**Table 1:** Countries, institutions, and authors ranked by publication volume and centrality.

Category	Rank	Country/Institutes/Author	Publications	Country/Institutes/Author	Centrality
Country Rank	1	USA	242(23.18%)	USA	0.63
	2	Peoples R China	182(17.43%)	England	0.21
	3	Italy	54(5.17%)	France	0.14
	4	Spain	41(3.92%)	Italy	0.12
	5	Australia	38(3.63%)	Australia	0.11
	6	France	35(3.35%)	Spain	0.1
	7	Canada	33(3.16%)	Iran	0.1
	8	England	31(2.96%)	Germany	0.08
	9	Japan	30(2.87%)	Peoples R China	0.07
	10	Germany	28(2.68%)	Saudi Arabia	0.06
Institute Rank	1	University of Helsinki	14	University of Helsinki	0.07
	2	Harvard University	13	National Institutes of Health	0.05
	3	Sun Yat Sen Univ	10	Fred Hutchinson Cancer Center	0.04
	4	Sun Yat-Sen University	10	Harvard University	0.03
	5	National Institutes of Health	9	The University of North Carolina	0.03
	6	University of Illinois	9	University System of Maryland	0.02
	7	Sichuan University	9	University Of Washington	0.02
	8	The University of Hong Kong	8	Boston Children’s Hospital	0.02
	9	University of Debrecen	8	King’s College London	0.01
	10	China Medical University	8	University of Oslo	0.01
Author Rank	1	Adlercreutz, H	10	Adlercreutz, H	0.01
	2	Bai, Peter	9	/	/
	3	Goedert, James J	4	/	/
	4	Miko, Edit	8	/	/
	5	Ujlaki, Gyula	8	/	/
	6	Tagliabue, Elda	3	/	/
	7	Kiss, Borbala	4	/	/
	8	Arkosy, Peter	3	/	/
	9	Kovacs, Tunde	4	/	/
	10	Sebo, Eva	4	/	/

Table 1 and Figure 3B highlight the top 10 contributing institutions among the 447 identified in this field. The leading institutions in terms of publication output are The University of Helsinki, Harvard University, Sun Yat-Sen University, The University of Alabama System, Sichuan University, the National Institutes of Health, the University of Illinois, the University of Debrecen, The University of Hong Kong, and China Medical University. However, when considering centrality, the top 10 institutions are the University of Helsinki, the National Institutes of Health, the Fred Hutchinson Cancer Center, Harvard University, The University of North Carolina, the University System of Maryland, the University of Washington, Boston Children’s Hospital, King’s College London, and the University of Oslo. None of the Chinese research institutions are included in the top 10 based on centrality.

In Table 1 and Figure 3C, the top 10 contributing authors out of the 612 authors in this field are presented. Adlercreutz H, Bai Peter, Goedert James J, Miko Edit, Ujlaki Gyula, Tagliabue Elda, Kiss Borbala, Arkosy Peter, Kovacs Tunde, and Sebo Eva have made significant contributions. Notably, Adlercreutz H stands out as the only author with a centrality value of 0.01.

### 3.3 Analysis of highly cited references and burst

The top 10 highly cited articles in this field have been published in prestigious journals. Three of these articles, published in *Science*, specifically focus on the impact of gut microbiome on the effectiveness of PD-1 immunotherapy. Another article investigates the role of gut microbiome metabolites in cancer development. The remaining six articles delve into the classification of breast cancer subtypes of breast cancer through microbial signatures (Figure 1A; Table 2).

**Table 2:** Top 10 highly cited references.

Rank	Citation	Year	Title	Source
1	87	2018	Gut microbiome influences the efficacy of PD-1-based immunotherapy against epithelial tumors	Science
2	81	2018	Gut microbiome modulates response to anti-PD-1 immunotherapy in melanoma patients	Science
3	77	2018	Breast cancer in postmenopausal women is associated with an altered gut metagenome	Microbiome
4	76	2018	Breast cancer and its relationship with the microbiota	Int J Env Res Pub He
5	62	2020	The human tumor microbiome is composed of tumor-type-specific intracellular bacteria	Science
6	55	2018	Distinct microbial signatures associated with different breast cancer types	Front Microbiol
7	54	2018	The commensal microbiome is associated with anti-PD-1 efficacy in metastatic melanoma patients	Science
8	52	2019	Preexisting commensal dysbiosis is a host-intrinsic regulator of tissue inflammation and tumor cell dissemination in hormone receptor-positive breast cancer	Cancer Res
9	49	2019	Microbiome-microbial metabolome–cancer cell interactions in breast cancer-familiar, but unexplored	Cells-Basel
10	45	2018	Lithocholic acid, a bacterial metabolite reduces breast cancer cell proliferation and aggressiveness	BBA-Bioenergetics

**Figure 4:**
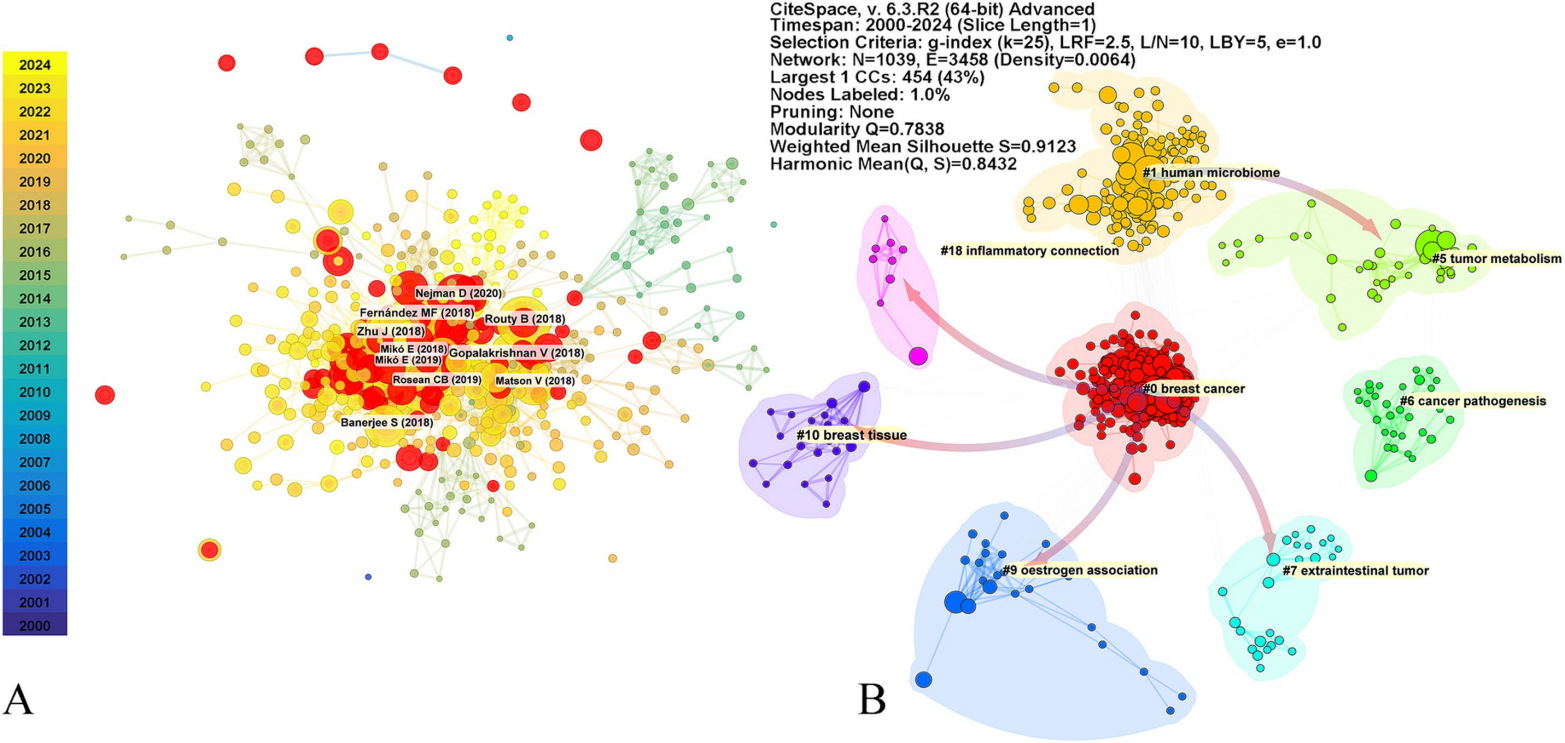
Top 10 cited reference and reference cluster. The top 10 cited references **(A)**, and the reference bursts with a minimum duration of one are colored in red. The nodes with K > 1 were clustered into different clusters **(B)**. The color arrows represent reference dependencies. The parameters of the CiteSpace analysis are displayed in the top right corner of the figure.

These articles are categorized into various clusters, including #0 “breast cancer,” #1 “human microbiome,” #5 “tumor metabolism,” #6 “cancer pathogenesis,” #7 “extraintestinal tumor,” #9 “estrogen association,” #10 “breast tissue,” and #18 “inflammatory connection.” The latest research bursts continue to focus on breast cancer and the human microbiome (Figure 4). Reference bursts, which indicate rapid growth in citations within a short timeframe, serve as indicators of emerging research hotspots and future directions (Table 3). The article titled “Investigation of the association between the fecal microbiome and breast cancer in postmenopausal women: a population-based case–control pilot study” holds the highest burst score. This article focuses on immunotherapy, while three other articles explore breast tissues and the intratumoral microbiome. Additionally, three studies focus on characterizing different subtypes of breast cancer microbiome. The citation network indicates that research on the microbiome’s relationship with breast cancer has expanded to encompass diverse topics such as “inflammatory connection,” “breast tissue,” “oestrogen association,” “extraintestinal tumor,” and “tumor metabolism.”

**Table 3:** Top 10 cited references burst.

Burst score	BurstBegin	BurstEnd	Title	Source
17.91	2017	2020	Investigation of the association between the fecal microbiota and breast cancer in postmenopausal women: a population-based case–control pilot study	J Natl Cancer Inst
12.89	2019	2021	The microbiota of breast tissue and its association with breast cancer	Appl Environ Microbiol
12.44	2018	2021	The intestinal microbiome and estrogen receptor-positive female breast cancer	J Natl Cancer Inst
11.57	2017	2020	Commensal Bifidobacterium promotes antitumor immunity and facilitates anti-PD-L1 efficacy	Science
11.57	2017	2020	Anticancer immunotherapy by CTLA-4 blockade relies on the gut microbiota	Science
10.77	2017	2021	The microbiome of aseptically collected human breast tissue in benign and malignant disease	Sci Rep
9.25	2019	2021	Characterization of the microbiome of nipple aspirate fluid of breast cancer survivors	Sci Rep
8.76	2017	2019	Associations of the fecal microbiome with urinary estrogens and estrogen metabolites in postmenopausal women	J Clin Endocrinol Metab
8.43	2022	2024	The human tumor microbiome is composed of tumor type-specific intracellular bacteria	Science
8.38	2022	2024	Intestinal microbiota influences clinical outcome and side effects of early breast cancer treatment	Cell Death Differ

### 3.4 Analysis of keywords co-occurrence and cluster

Table 4 presents the top 10 keywords in terms of co-occurrence rate and centrality in the research field of microbiome and breast cancer from 2000 to 2024. The most frequently occurring keywords are “breast cancer” and “gut microbiome,” followed by “risk,” “breast cancer cells,” “colorectal cancer,” “cells,” “expression,” “metabolism,” “bacteria,” “short-chain fatty acids,” and “inflammation.” Centrality, which measures the degree of connection between keywords, positively correlates with the importance of a keyword in the research network.

**Table 4:** Frequency and centrality of top 25 used keywords.

Rank	Frequency	Keyword	Centrality	Keyword
1	440	breast cancer	0.42	breast cancer
2	403	gut microbiome	0.31	gut microbiome
3	127	risk	0.21	risk
4	86	colorectal cancer	0.19	breast cancer cells
5	49	cells	0.13	colon cancer
6	43	metabolism	0.09	colorectal cancer
7	43	expression	0.09	bacteria
8	40	bacteria	0.08	Cells
9	39	chain fatty acids	0.08	*in vitro*
10	38	inflammation	0.05	metabolism
11	36	health	0.05	chain fatty acids
12	36	association	0.05	postmenopausal women
13	36	obesity	0.05	apoptosis
14	33	breast cancer cells	0.04	expression
15	30	postmenopausal women	0.04	growth
16	30	oxidative stress	0.04	body mass index
17	29	colon cancer	0.04	activation
18	29	chemotherapy	0.03	health
19	28	cancer	0.03	oxidative stress
20	25	growth	0.03	chemotherapy
21	24	*in vitro*	0.03	women
22	23	women	0.03	therapy
23	22	therapy	0.03	estrogen
24	21	body mass index	0.03	tumor microenvironment
25	21	activation	0.03	diet

In terms of co-occurrence rate, the most frequently cited keywords remain “breast cancer,” “gut microbiome,” “risk,” and “breast cancer cells.” They are followed by “colon cancer,” “colorectal cancer,” “bacteria,” “*in-vitro*,” “cells,” and “apoptosis.” The notable rise of keywords related to *in vitro* studies highlights the current focus and topic within the field.

**Figure 5:**
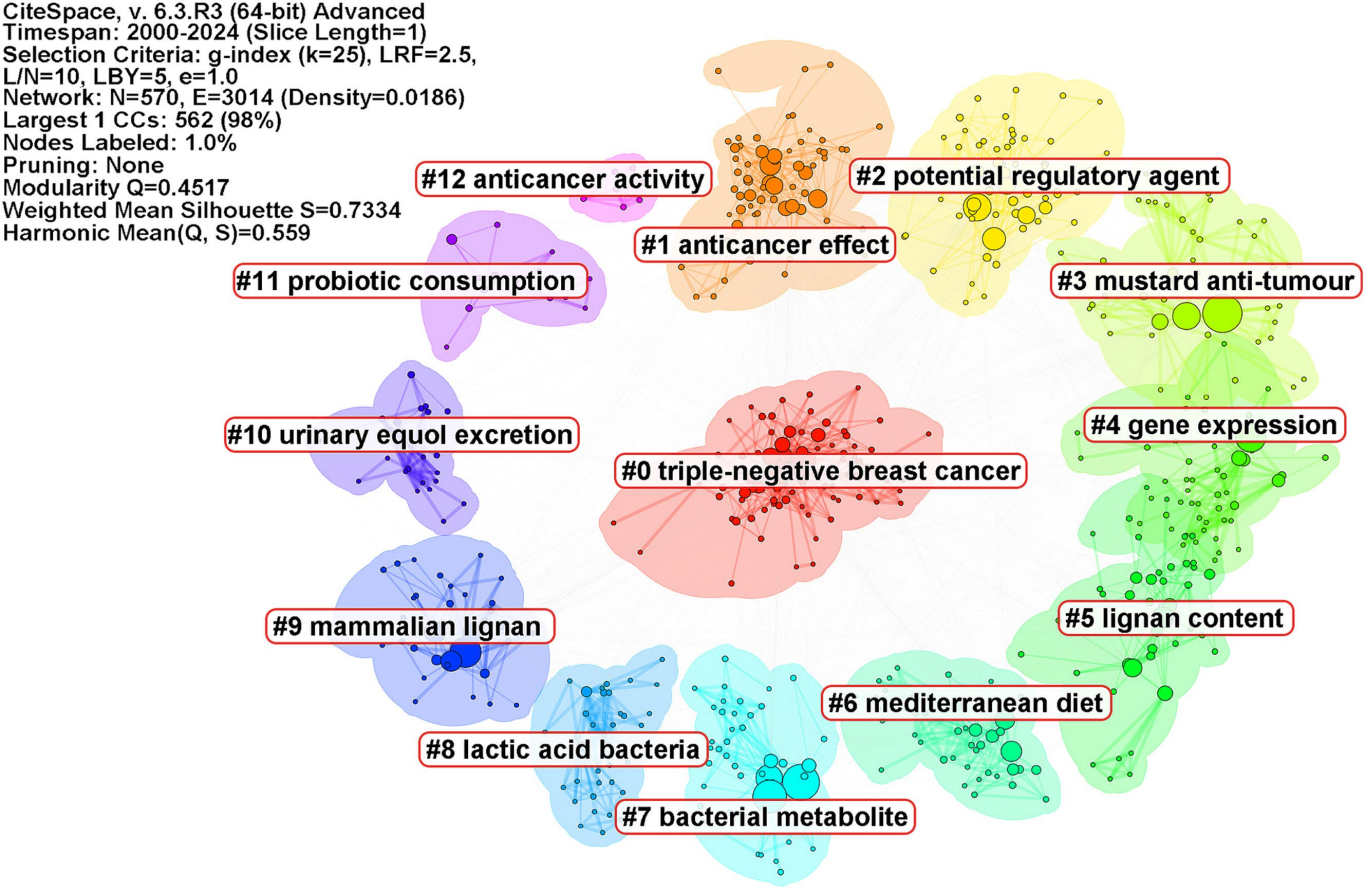
Keywords clustering map. Keywords were clustered and visualized using a circular layout. Different colors represent distinct clusters, with cluster names identified using the log-likelihood ratio method labeled accordingly. The parameters of the CiteSpace analysis are displayed in the top right corner of each figure.

To gain further insights into the research frontiers of microbiome and breast cancer, the keywords were clustered using CiteSpace (Figure 5). Cluster #0 is named “triple-negative breast cancer,” followed by cluster #1 “anticancer effect,” cluster #2 “potential regulatory agent,” cluster #3 “mustard anti-tumor,” cluster #4 “gene expression,” cluster #5 “lignan content,” cluster #6 “Mediterranean diet,” cluster #7 “bacterial metabolite,” cluster #8 “lactic acid bacteria,” and cluster #9 “mammalian lignan.” These clusters collectively indicate that researchers are investigating the potential of the microbiome to influence cancer development, treatment, and personalized therapeutic approaches.

## 4 Discussion

### 4.1 Information overview discussion

We collected all quality data from WoSCC to illustrate the research frontiers and hot topics in microbiome and breast cancer studies. A total of 736 publications matched our research criteria, showing a consistent growth trend in publication volume. The geographic distribution of publications reveals that the United States leads in publication output, followed by China and Italy, reflecting a concentrated research effort on microbiome and breast cancer in these countries. However, while China ranks second in publication volume, it is ninth in centrality, indicating relatively weaker international collaboration and influence compared to other leading countries. A similar trend was observed in the analysis of institutional contributions. Furthermore, the top 10 contributing authors all come from Western countries, indicating a potential gap between publication quantity and research impact or quality in China. Among these authors, only one author had a notable centrality value, suggesting the need to strengthen cooperative relationships among countries, institutions, and researchers to advance this scientific field effectively.

### 4.2 Research hotspot and frontier

The references and keyword analysis reveal the primary research focus on the interplay between the microbiome and breast cancer. Key hotspots include exploring microbiome-based breast tumor metabolism and the microbiome’s characteristics that potentially increase breast cancer risk, as indicated by keywords such as “cancer pathogenesis,” “tumor metabolism,” “risk,” and “metabolism.” Furthermore, keyword bursts and clustering results indicate that research frontiers are moving toward anti-tumor therapies and personalized medicine, with significant terms including “anti-tumor,” “potential regulatory agent,” and “Mediterranean diet.” Notably, among the top 10 cited references, studies on intratumor bacteria in breast tissue are emerging as a significant area of interest (Chlebowski et al., 2024).

### 4.3 The interplay between the microbiome and breast cancer can exhibit an intriguing anti-tumor effect

The anti-tumor effect of the microbiome was frequently mentioned in our bibliometrics analysis. Emerging research suggests that specific microbes within the microbiome can exert anti-tumor effects in breast cancer.

The microbiome interacts with the immune system, modulating its functionality and response to cancer cells (Parida et al., 2021). Certain bacteria have been found to stimulate immune cells and enhance anti-tumor immune responses, leading to the inhibition of tumor growth and metastasis (Parhi et al., 2020; Nakkarach et al., 2021). Nisin, a bacteriocin produced by various species of *Lactobacillus* and *Streptococcus* during fermentation, has shown promise. It is widely regarded as safe for human use and has been found to induce apoptosis in cancer cell lines such as MCF-7 cells (derived from human mammary adenocarcinoma) and HT-29 cells (derived from human colon cancer) (Khazaei Monfared et al., 2022). Phenazine metabolites, secreted by strains of *Pseudomonas aeruginosa* and other bacterial species such as *Streptomyces* sp., exhibit cytotoxic activity on tumor cell lines. Specifically, phenazine 1,6-dicarboxylic acid has demonstrated a substantial impact on the viability of HeLa, HT-29, and MCF-7 cell lines (Dasgupta et al., 2015). On the other hand, dysbiosis, an imbalance in the microbial community contributes to chronic inflammation, a critical factor in cancer development and progression (Yue et al., 2023; Yu et al., 2023). By promoting a healthy and diverse microbiome, inflammation can be reduced, potentially limiting the growth and spread of breast cancer cells (Parida and Sharma, 2019).

The microbiome can significantly influence the metabolism of estrogen and other hormones, which are crucial in the development of both estrogen receptor (ER)-dependent and ER-independent breast cancer (Hou et al., 2021; Ervin et al., 2019). The term “estrobolome” specifically refers to the collection of bacterial genes within the gut microbiome that can metabolize estrogens (Plottel and Blaser, 2011). The disruption of the microecological balance can result in an imbalance of estrogen reactivation and inactivation, ultimately leading to overexposure of target organs to estrogen or affecting drug efficacy (Plottel and Blaser, 2011). Notably, antibiotic use has been shown to impact estrogen levels by affecting the concentrations of conjugated and de-conjugated estrogens within the gut. Research from as early as the last century indicated that ampicillin consumption could increase fecal-conjugated estrogens in women (Adlercreutz et al., 1976; Martin et al., 1975). This suggests that modulating the gut microbiome could be a potential strategy to influence the half-life and bioavailability of these hormonal agents (Chen et al., 2018), opening new avenues for managing hormone-dependent breast cancer.

The composition of the microbiome has been linked to the response to breast cancer treatments, including chemotherapy and immunotherapy (Li et al., 2023; Cammarota et al., 2020). Certain beneficial bacteria have been associated with improved treatment outcomes and enhanced the efficacy of specific therapies (Cammarota et al., 2020; Bawaneh et al., 2022). For example, *Bacteroides fragilis* (*B. fragilis*) was demonstrated to contribute to oxaliplatin efficacy by stimulating the infiltration of CD8^+^ cytotoxic T lymphocytes into the tumor microenvironment (Zhu et al., 2021). In patients who received capecitabine, those with shorter progression-free survival (PFS) were characterized by a gut microbial composition dominated by *Slackia*. In contrast, patients with *Blautia obeum* exhibited significantly prolonged PFS compared to those without this microbial presence (Guan et al., 2020). Patients who did not respond (NR) to trastuzumab exhibited lower *α* diversity and reduced abundance of *Lachnospiraceae*, *Turicibacteraceae*, *Bifidobacteriaceae*, and *Prevotellaceae* compared to those who achieved pathologic complete response (CR). These observations were further validated in fecal microbiome transplantation (FMT) experiments in HER2-positive breast cancer mouse models, where gut dysbiosis in NR mice impaired the recruitment of CD4^+^ T cells and granzyme B-positive cells. In contrast, the gut microbiome of CR patients positively correlated with an enhanced immune response, including increased levels of interferon, NO2-IL12, and activated CD4^+^ T cells and dendritic cells (DCs) in tumors (di Modica et al., 2021).

While there is compelling evidence that the microbiome can support anti-cancer therapies, the translation from laboratory findings to clinical applications presents significant challenges. Future research should focus on understanding the precise mechanisms through which the gut microbiome influences breast cancer progression and treatment response. This may include identifying key species and their metabolites that exert anti-tumor effects and exploring how these can be harnessed or modulated to improve therapeutic outcomes. Personalized microbiome-based interventions, such as probiotics, prebiotics, antibiotics, or dietary modifications, could potentially enhance the efficacy of existing breast cancer treatments. Additionally, the advantages of artificial intelligence (AI) in integrating multidimensional data could facilitate the incorporation of microbiome research into breast cancer management, enabling precision medicine approaches tailored to individual patients.

### 4.4 The microbiome interplay with breast cancer by diet

The interplay between the microbiome and breast cancer can be influenced significantly by diet as dietary habits can significantly shape the gut microbiome and influence breast cancer risk, progression, and treatment responses (Arnone et al., 2024; Yue et al., 2023). Numerous studies emphasize that diet modulates the microbiome–breast cancer interaction by altering microbial composition and activity.

A diet high in fiber and plant-based foods fosters a diverse and healthy microbiome by promoting the growth of beneficial bacteria, which ferment dietary fiber into short-chain fatty acids (SCFAs) such as butyrate and propionate. SCFAs exhibit anti-inflammatory properties that may reduce breast cancer risk (Song et al., 2022; Park et al., 2021). *Escherichia coli* KUB-36 (*E. coli* KUB-36) isolated from human fecal samples can produce seven different SCFAs *in vitro*, which show cytotoxic effects on MCF-7 breast cancer cells and an anti-inflammatory response in LPS-stimulated THP-1 macrophages. Interestingly, impaired SCFA production by the gut microbiome has been observed in premenopausal breast cancer patients, including reduced levels of propionate and butyrate. Sodium propionate demonstrates anticancer effects by modulating the JAK2/STAT3/ROS/p38 MAPK signaling pathway in breast cancer xenografts in mice. Particularly, the #6 “Mediterranean diet” cluster represents a high-fiber diet that has been reported to have a protective effect on the risk of breast cancer (Pellegrini et al., 2020; González-Palacios Torres et al., 2023). A study involving 552 participants demonstrated that a higher Mediterranean diet index was associated with a reduced risk of breast cancer development through the modulation of chronic low-grade inflammation (Quartiroli et al., 2024).

Consuming high-fat diets (HFDs), characterized by a high intake of processed foods, red meat, and saturated fats, is linked to an unfavorable microbiome composition. Compared to the Mediterranean diet, such diets may promote the growth of harmful bacteria and decrease the abundance of beneficial ones (Malesza et al., 2021). This dysbiosis can contribute to an impaired gut barrier and chronic inflammation, which is linked to an increased risk of breast cancer (Nagahashi et al., 2018; Clayburgh et al., 2005). In mice mode, both HFDs and fecal microbiome derived from HFD-fed mice reduced the expression of tight junction-associated genes in the gut and mammary glands, along with elevated bacterial lipopolysaccharide levels in serum. Moreover, the HFD-derived microbiome exhibited the ability to increase breast cancer cell proliferation and implicated tumor-associated bacteria in cancer signaling (Soto-Pantoja et al., 2021). Additionally, leucine from the HFD microbiome activates the mTORC1 pathway in myeloid progenitors, increasing polymorphonuclear myeloid-derived suppressor cell (PMN-MDSC) differentiation, which is associated with tumoral PMN-MDSC infiltration and poorer outcomes in breast cancer patients (Chen et al., 2024).

Given diet’s central role in shaping the microbiome, identifying dietary components that promote a beneficial microbiome could aid breast cancer prevention and treatment. Investigations into dietary patterns such as the Mediterranean diet could inform guidelines to support breast cancer patients’ microbiome health. Artificial intelligence could further personalize these nutritional strategies by analyzing individual microbiome compositions, potentially enhancing treatment effectiveness and improving clinical outcomes for breast cancer patients.

### 4.5 The microbiome interplay with breast cancer by lignan

Lignans, a class of naturally occurring polyphenols found in plants, are frequently cited for their diverse health benefits in breast cancer. These compounds, categorized as phytoestrogens, exhibit estrogen-like structures and functions, albeit with much lower potency compared to endogenous estrogens (Lephart, 2021).

Lignans are widely found in various plants, especially in seeds (such as flaxseeds and sesame seeds), whole grains, legumes, fruits, and vegetables. They have been characterized as having antioxidant, anti-inflammatory, anti-menopausal, and anticancer effects in many studies (Jang et al., 2022). These functions are realized through the modulation of various mediators such as reactive oxygen species (ROS), nuclear factor erythroid 2-related factor 2 (Nrf2), cyclooxygenases (COXs), TNF-*α*, IL-1β, COX-2, and iNOS, which can be affected by the supplementation of lignan-rich foods (Jang et al., 2022; Li et al., 2023; Clavel et al., 2006). Most of the biological activity of lignans is obtained after metabolism by the gut microbiome. For example, secoisolariciresinol is demethylated or dehydroxylated by *Peptostreptococcus productus* and *Eggerthella lenta* to produce enterodiol and enterolactone metabolites, which are readily absorbed into the bloodstream from the gut (Clavel et al., 2006). Another study suggested that not secoisolariciresinol diglucoside showed no negative effects on the MCF-7 cell but the derivatives of secoisolariciresinol and secoisolariciresinol-4′,4″-diacetate (Scherbakov et al., 2021). The flaxseed lignan secoisolariciresinol diglucoside was demonstrated to reduce tumor growth in the E0771 TNBC mice model, likely by inhibiting NF-κB activity (Bowers et al., 2019). Flaxseed is one of the richest dietary sources of lignans and has been reported to mediate mammary gland miRNA expression with lower PI3K-Akt–mTOR pathway-related gene expression through the alteration of the cecal microbiome (Wu et al., 2024).

Given their diverse mechanisms of action, lignans and their derivatives hold significant potential for anticancer drug development. Many lignans have demonstrated specific anti-breast cancer activities. For instance, arctigenin has been reported to inhibit proliferation and induce apoptosis in breast cancer cells *in vitro* by targeting the transcription factor STAT3 (Feng et al., 2017). Another study demonstrated that arctigenin exhibits anti-metastatic effects on breast cancer cell lines by inhibiting MMP-9 and uPA by targeting the Akt, NF-κB, and MAPK signaling pathways (Maxwell et al., 2017). The role of the gut microbiome is crucial in converting dietary lignans into bioactive forms, enhancing their anticancer efficacy. For example, arctigenin is metabolized by human gut bacteria into bioactive metabolites, including arctiin and enterolactone, which are better absorbed and may exhibit improved therapeutic activity (Xie et al., 2003). Another lignan derivative, (−)-hinokinin, is a dibenzylbutyrolactone lignan synthesized from (−)-cubebin extracted from *Piper cubeba* seeds. This compound has shown potential in inducing G2/M cell cycle arrest and apoptosis in MCF-7 breast cancer cells and exhibits synergistic effects with doxorubicin in inhibiting SKBR-3 breast cancer cell proliferation by modulating cyclin-dependent kinase inhibitor 1 (Cunha et al., 2016). However, many lignans require specific structural modifications to optimize their bioactivity. Podophyllotoxin, a well-known lignan extracted from *Podophyllum peltatum*, exhibits strong antiviral and antimitotic properties, though its use has been limited due to high toxicity (Ardalani et al., 2017; Gordaliza et al., 2000). Subsequent research on podophyllotoxin derivatives led to the development of etoposide and teniposide, which have lower cytotoxicity and were approved by the FDA for clinical use as ETOPOPHOS® and TENIPOSIDE® (Newman and Cragg, 2020), respectively. These drugs are now used to treat lymphomas, non-lymphocytic leukemia, and other cancers, often in combination with other chemotherapy agents.

Although lignans have lower potency than human hormones, their broad availability in dietary sources and the cumulative impact of long-term exposure may have significant, yet underappreciated, health benefits. Conducting long-term, longitudinal studies to fully understand the effects of sustained lignan exposure is essential. Additionally, adjusting both the quantity and types of lignans consumed through dietary interventions represents a promising, innovative approach to breast cancer prevention and intervention. This strategy could offer a non-invasive, lifestyle-based option to modulate breast cancer risk, particularly by harnessing lignans’ hormone-modulating, antioxidant, and anti-inflammatory properties over time.

### 4.6 The relationship between breast microbiome and breast cancer: emerging research

Breast tissue is typically considered sterile; however, the microbiome of breast tissue has attracted the attention of researchers after it was recognized that the microbiome occurs in breast milk and that microbes may enter the breast tissue interior through the breast ducts (Urbaniak et al., 2014; Meng et al., 2018; Tzeng et al., 2021). Studies on gut microbiomes have demonstrated that these microbes may intervene in shaping the occurrence and development of diseases through various mechanisms (Chang and Pekkle Lam, 2024). The precise mechanisms by which intracellular bacteria affect tumor cells and influence breast cancer progression remain unclear. However, current research suggests several potential mechanisms: (i) DNA damage, (ii) promotion of epithelial–mesenchymal transition (EMT) and proliferation, and (iii) antitumor immunity.

#### 4.6.1 DNA damage

DNA damage is a critical factor in breast cancer development, as it disrupts the normal function of genes that regulate cell growth, repair, and survival. In many cases, breast cancer initiates when DNA in breast cells accumulates mutations or damage over time (Alhmoud et al., 2020). Studies show that *E. coli* is more abundant in breast cancer patients compared to healthy controls, and *E. coli* isolated from tumor-adjacent tissues has been shown to induce DNA double-stranded breaks (Urbaniak et al., 2016). Additionally, toxins from *B. fragilis* and *Campylobacter jejuni* can cause DNA damage through the production of reactive oxygen species (ROS), a mechanism known to drive colon tumorigenesis (Goodwin et al., 2011). Nevertheless, this bacterial DNA-damaging effect also plays a role in enhancing the efficacy of antitumor drug therapy. Platinum-based compounds, such as oxaliplatin and cisplatin, induce DNA double-strand breaks by forming intrastrand platinum-DNA adducts, which inhibit DNA replication. However, germ-free or antibiotic-treated mice fail to respond to oxaliplatin or cisplatin, highlighting the importance of microbial presence in drug efficacy (Iida et al., 2013). Concurrent use of probiotics, such as *Lactobacillus acidophilus*, has been shown to restore cisplatin-induced gene expression and improve cisplatin’s therapeutic response (Gui et al., 2015).

#### 4.6.2 Promotion of EMT and proliferation

EMT and cellular proliferation are essential processes in breast cancer progression. EMT is a biological program where epithelial cells lose their polarity and adhesion properties, adopting mesenchymal characteristics that enable them to migrate, invade, and resist apoptosis. This transition is a crucial mechanism by which cancer cells acquire metastatic potential, spreading from the primary tumor to distant sites (Chen et al., 2017). Bacteria-induced DNA damage alone may not be sufficient to cause cancer but can become detrimental in combination with other risk factors. For instance, increased metabolic conversion of progesterone to 5-alpha-3,20-dione (5αP) has been observed in breast tumors compared to normal tissue; this metabolite can induce breast cancer cell proliferation *in vitro*. *Bacillus cereus* exhibits this metabolic activity, and its presence has been noted to increase in breast cancer tissue (Urbaniak et al., 2016; Filippone et al., 2023).

Furthermore, antibiotic treatment has been shown to hinder breast cancer metastasis, while re-administration of bacteria leads to increased lung metastasis (Fu et al., 2022). These intracellular bacteria may promote cancer cell metastasis through the suppression of RhoA and ROCK activation, and the inhibition of ROCK or antibiotic treatment negates the viability changes caused by bacterial invasion (Fu et al., 2022). In other research, *B. fragilis* has been found to colonize breast cancer tissue, and colonization of enterotoxigenic *B. fragilis* in the gut or mammary gland has been shown to induce the growth and metastasis of cancer cells. The presence of this intracellular bacteria within breast cancer cells may further influence cancer metastasis. It appears that the Notch1 and Wnt-*β*-catenin signaling pathways are involved in this process; inhibiting Notch and β-catenin reduces *B. fragilis* toxin-mediated migration and invasion of breast cells (Parida et al., 2021). A comprehensive analysis revealed that *H. influenza* was observed to correlate with genes responding to breast cancer growth, such as G2M checkpoint, E2F signaling, and mitotic spindle assembly (Thompson et al., 2017).

#### 4.6.3 Antitumor immunity

The bacteria are generally considered immunostimulatory due to their foreign antigens, the impact of specific intracellular bacteria on breast cancer immunity varies with the bacterial species, location, and interactions with the host immune system. Intracellular bacteria express pathogen-associated molecular patterns (PAMPs) that bind to pattern recognition receptors (PRRs), such as Toll-like receptors (TLRs) and NOD-like receptors (NLRs). This interaction triggers the release of proinflammatory cytokines, which attract and activate immune cells in the tumor microenvironment, potentially promoting an immune response against cancer cells. Dysbiosis in the breast microbiome can lead to inflammation in breast tissue, increasing M2-like macrophage infiltration, enhancing levels of interleukin (IL)-23, IL-22, CXCL-1, and CCL-2, and accelerating fibrosis within the tumor environment (Buchta Rosean et al., 2019). Compared to healthy controls, breast tissue also shows significant downregulation of TLR4, alongside upregulation of MYD88, IRAK1, and other downstream genes, suggesting altered immune signaling pathways (Tzeng et al., 2021). When intracellular bacteria infect antigen-presenting cells (APCs), their antigens are presented on major histocompatibility complex (MHC) molecules, enhancing the activation of cytotoxic T-cells. This response may also promote the immune system’s recognition of tumor-associated antigens, leading to a more robust antitumor immune response. For example, breast tumors with an activated immune microenvironment include a higher abundance of *Clostridiales*. Notably, some genera within *Clostridiales* produce trimethylamine (TMA), the precursor of trimethylamine N-oxide (TMAO). TMAO has been shown to enhance antitumor immunity in TNBC by activating the endoplasmic reticulum stress kinase PERK pathway, thus promoting CD8^+^ T cell-mediated immunity and ultimately leading to tumor cell pyroptosis. Clinical samples have confirmed that TMAO levels correlate positively with immunotherapy efficacy, highlighting the potential therapeutic relevance of this metabolite (Wang et al., 2022). Previous studies indicated that dysbiosis of the breast microbiome may lead to the occurrence of mastitis, characterized by an increase in microbial load and the promotion of IL-8 secretion by mammary epithelial cells (Boix-Amorós et al., 2020). In addition, *Fusobacterium nucleatum* (*F. nucleatum*), commonly found in colorectal cancer and associated with poor treatment outcomes, has also been observed in breast cancer. In breast cancer models, intravenous injection of Fap2-expressing *F. nucleatum* ATCC 23726 resulted in reduced tumor-infiltrating T cells and promoted tumor growth and metastasis. This immunosuppressive effect, however, could be mitigated with antibiotic treatment, illustrating the potential impact of bacterial presence on immune evasion and tumor progression in breast cancer (Parhi et al., 2020).

The breast microbiome is much less abundant than the gut microbiome, making it more challenging to study, and research in this field is still in its early stages. Understanding the interplay between microbiome and tumors holds great promise and is emerging as a current research hotspot.

### 4.7 Future directions and challenges

While research on the microbiome and breast cancer has made significant progress, there are still many challenges and future directions to explore:

- Standardization and reproducibility: there are many studies reported on the interaction between microbiome and breast cancer. However, comparing results across different studies is challenging due to the variations in sampling, sequencing, and analysis methods. These inconsistencies make it difficult to enable more robust conclusions and establish consistency across studies. To address this issue, the adoption of standardized methods and operational processes is crucial. Implementing standard protocols for sampling, sequencing, data analysis, and result interpretation will enhance the reliability and comparability of research outcomes.
- Causality and mechanistic understanding: while many studies have identified associations between the microbiome and breast cancer, establishing causality and understanding the underlying mechanisms is essential. To further investigate the causal relationships between the microbiome and breast cancer, it is crucial to integrate artificial intelligence technologies and conduct more long-term longitudinal studies, clinical trials, and animal experiments. These efforts will contribute to leveraging the insights gained from the microbiome perspective and can potentially provide innovative solutions for addressing the development, progression, and treatment response of breast cancer.
- Personalized approaches: the microbiome varies considerably between individuals. With rapid advances in sequencing technology, the field of microbiome research is moving toward personalized medicine, and this approach should be extended to breast cancer. Differentiated, targeted interventions based on individual differences in the microbiome, as well as individual differences in breast cancer, will enable personalized treatment, including dietary modifications, probiotics, and targeted therapies.
- Therapeutic interventions: intervention in breast cancer prevention and treatment by manipulating the microbiome is an exciting potential avenue for future research. Identifying specific beneficial bacteria, or bacterial products, that can regulate tumor growth, improve treatment response, and minimize side effects is an active area of research. In addition, it is a great challenge to study how to regulate gut bacteria at the guild functional level, whether through phage or gene editing technology.
- International collaboration: strengthening international collaboration and knowledge exchange will help bridge the gap between countries and research institutions. Encouraging interdisciplinary collaborations between microbiome researchers, oncologists, immunologists, and nutritionists will lead to a more comprehensive understanding of the microbiome–breast cancer interaction.

## 5 Advantages and limitations

Bibliometric analysis provides a comprehensive overview of the research landscape by identifying and analyzing publications related to the interaction between the microbiome and breast cancer. It helps in understanding the growth, trends, and key contributors in the field. It helps researchers focus on unexplored aspects of the microbiome–breast cancer interaction, thereby guiding future research directions. However, this analysis is limited to the analysis of published articles in WoSCC, which may exclude publication in other databases and non-English publications. This may result in an incomplete picture of the microbiome–breast cancer interaction.

## 6 Conclusion

The publication on the microbiome and breast cancer was evaluated and visualized in our study. The research in this field is continuously active and continues to increase the number of publications each year. The United States stands out as the leading contributor in terms of publications on the interaction between the microbiome and breast cancer, showing its significant research output and expertise in the field. However, an interesting observation is the potential gap between publication quantity and research impact or quality in China, suggesting an area of opportunity for further investigation and improvement. The University of Helsinki and Adlercreutz H are the most influential institutions and researchers in the field. Recent research centered on the exploration of microbiome-based breast tumor metabolism and the research frontiers were hit on anti-tumor therapies and personalized medicine. At the same time, the intertumoral microbiome research may be on the rise.

## Data Availability

The original contributions presented in the study are included in the article/Supplementary material, further inquiries can be directed to the corresponding authors.
