# Supplementary material for: Bibliometric and visual analysis of human microbiome—breast cancer interactions: current insights and future directions
Source: Front Microbiol. 2024 Dec 9;15:1490007. doi: 10.3389/fmicb.2024.1490007 (PMC11664440; doi:10.3389/fmicb.2024.1490007)
Supplement: Supplementary file 1 [file Table_1.DOCX]

TS = ((“Gastrointestinal Microbiome*” OR “Microbiome*, Gastrointestinal” OR “Gut Microbiome*” OR “Microbiome*, Gut” OR “Gut Microflora” OR “Microflora, Gut” OR “Gut Microbiota*” OR “Microbiota*, Gut” OR “Gastrointestinal Flora” OR “Flora, Gastrointestinal” OR “Gut Flora” OR “Flora, Gut” OR “Gastrointestinal Microbiota*” OR “Microbiota*, Gastrointestinal” OR “Gastrointestinal Microbial Communit*” OR “Microbial Communit*, Gastrointestinal” OR “Gastrointestinal Microflora” OR “Microflora, Gastrointestinal” OR “Gastric Microbiome*” OR “Intestinal Microbiome*” OR “Microbiome, Gastric” OR “Microbiome, Intestinal” OR “Intestinal Microbiota*” OR “Microbiota, Intestinal” OR “Intestinal Microflora” OR “Microflora, Intestinal” OR “Intestinal Flora” OR “Flora, Intestinal”) AND (“Breast Cancer” OR “Cancer, Breast” OR “Breast Carcinoma*” OR “Carcinoma*, Breast” OR “Breast Neoplasm*” OR “Neoplasm*, Breast” OR “Mammary Cancer*” OR “Cancer*, Mammary” OR “Breast Carcinoma*” OR “Carcinoma*, Breast” OR “Mammary Neoplasm*” OR “Neoplasm*, Mammary”))
